# Generation of induced pluripotent stem cells from Bornean orangutans

**DOI:** 10.3389/fcell.2023.1331584

**Published:** 2024-01-05

**Authors:** Chia-Jung Li, Chia-Chun Chang, Li-Kuang Tsai, Min Peng, Wei-Ni Lyu, Jane-Fang Yu, Mong-Hsun Tsai, Li-Ying Sung

**Affiliations:** ^1^ Institute of Biotechnology, National Taiwan University, Taipei, Taiwan; ^2^ Department of Obstetrics and Gynecology, Chang Gung Memorial Hospital, Linkou Branch, Chang Gung University, Taoyuan, Taiwan; ^3^ Conservation and Research Center, Taipei Zoo, Taipei, Taiwan; ^4^ Center for Developmental Biology and Regenerative Medicine, Taipei, Taiwan; ^5^ Center for Biotechnology, National Taiwan University, Taipei, Taiwan; ^6^ Agricultural Biotechnology Research Center, Academia Sinica, Taipei, Taiwan

**Keywords:** Bornean orangutan, endangered species, induced pluripotent stem cell, reprogramming, pluripotency, differentiation

## Abstract

**Introduction:** Orangutans, classified under the Pongo genus, are an endangered non-human primate (NHP) species. Derivation of induced pluripotent stem cells (iPSCs) represents a promising avenue for conserving the genetic resources of these animals. Earlier studies focused on deriving orangutan iPSCs (o-iPSCs) from Sumatran orangutans (Pongo abelii). To date, no reports specifically target the other Critically Endangered species in the Pongo genus, the Bornean orangutans (*Pongo pygmaeus*).

**Methods:** Using Sendai virus-mediated Yamanaka factor-based reprogramming of peripheral blood mononuclear cells to generate iPSCs (bo-iPSCs) from a female captive Bornean orangutan. In this study, we evaluate the colony morphology, pluripotent markers, X chromosome activation status, and transcriptomic profile of the bo-iPSCs to demonstrate the pluripotency of iPSCs from Bornean orangutans.

**Results:** The bo-iPSCs were successfully derived from Bornean orangutans, using Sendai virus-mediated Yamanaka factor-based reprogramming of peripheral blood mononuclear cells. When a modified 4i/L/A (m4i/L/A) culture system was applied to activate the WNT signaling pathway in these bo-iPSCs, the derived cells (m-bo-iPSCs) manifested characteristics akin to human naive pluripotent stem cells, including high expression levels of KLF17, DNMT3L, and DPPA3/5, as well as the X chromosome reactivation. Comparative RNA-seq analysis positioned the m-bo-iPSCs between human naive and formative pluripotent states. Furthermore, the m-bo-iPSCs express differentiation capacity into all three germlines, evidenced by controlled *in vitro* embryoid body formation assay.

**Discussion:** Our work establishes a novel approach to preserve the genetic diversity of endangered Bornean orangutans while offering insights into primate stem cell pluripotency. In the future, derivation of the primordial germ cell-like cells (PGCLCs) from m-bo-iPSCs is needed to demonstrate the further specific application in species preservation and broaden the knowledge of primordial germ cell specification across species.

## 1 Introduction

Orangutans, one of humans’ closest relatives, have a 97.4% genome similarity to that of humans (Locke et al., 2011; Nakamura et al., 2021). The *International Union for Conservation of Nature* (IUCN) has listed all three orangutan species as Critically Endangered since 2008 Ancrenaz et al., 2016, including *Pongo pygmaeus*, *Pongo abelii*, and *Pongo tapanuliensis*.

In addition to conventional conservation strategies, modern biotechnologies, for example, induced pluripotent stem cell (iPSC) derivation and cryopreservation, offer new options to prevent their extinction by preserving their genetic resources (Saragusty et al., 2020; Voigt et al., 2022).

Limited work has been reported to derive orangutan iPSCs (o-iPSCs). In one work (Ramaswamy et al., 2015), a study generated o-iPSC from skin fibroblasts of captive Sumatran orangutans (*Pongo abelii*) through retroviral transduction of human *OCT4*, *SOX2, KLF4,* and *c-MYC* genes (*i.e.*, OSKM factors, also known as Yamanaka factors). Furthermore, several studies demonstrated that using a non-integration approach and combining easily accessible somatic cell sources could promise efficient and safe generation of iPSCs for further utilization and application in clinical settings (Dey et al., 2021; Ray et al., 2021). Building on the concept, in another work (Geuder and Lucas, 2021), o-iPSCs were derived from the urinary epithelial cells of Sumatran orangutans through the transfection of the Sendai virus, carrying three (i.e., Klf4-Oct3/4-Sox2) of the four OKSM factors.

Given the evolutional closeness between orangutans and humans, the generation of o-iPSCs would also contribute to our understanding of the evolutionary convergent and divergent processes involved in early embryo development across different primate species, especially humans. On the other hand, knowledge gained in human pluripotent stem cells (PSCs) may help improve the derivation process and the quality of PSCs of other NHP species, such as orangutans.

Human naive PSCs represent the ground state of pluripotency resembling the preimplantation epiblast, which offers greater and unbiased differentiation capacity than their primed pluripotent counterparts (Lee et al., 2017; Guo et al., 2021). Recent research has shed light on diverse methodologies for achieving the naive state *in vitro*, including converting primed pluripotency to a naive state using small-molecule cocktails (Guo et al., 2016; Theunissen et al., 2016; Bredenkamp et al., 2019; Rostovskaya, Stirparo, and Smith, 2019). Several culture systems have been reported to sustain self-renewal and evoke the acquisition of naive features in human PSCs, such as 5i/L/A (Theunissen et al., 2014), 4i/L/A (Theunissen et al., 2016), t2iLGÖ (Takashima et al., 2014), and PXGL (Bredenkamp et al., 2019). However, few studies have focused on regulating the pluripotency of NHP PSCs.

In this study, we present the generation of new iPSCs (bo-iPSCs) in another orangutan species, the Bornean orangutan, from the peripheral blood mononuclear cells (PBMCs) obtained from a captive female *Pongo pygmaeus* using the Sendai virus-mediated Yamanaka factor-based reprogramming method. We then subjected these bo-iPSCs to the modified 4i/L/A (m4i/L/A) culture system, which is known to promote the generation of naive state human pluripotent stem cells by activating the WNT pathway. The resultant modified bo-iPSCs (m-bo-iPSCs) exhibited key features akin to human naive stem cells and manifested differentiation capacity in an *in vitro* embryoid body formation assay. Our study provides insight into the molecular features and pluripotent signaling dependency of bo-iPSCs and presents a new option for Bornean orangutan conservation.

## 2 Materials and methods

### 2.1 Animals

All aspects of animal maintenance, care, and procedures were rigorously reviewed and granted ethical approval by the Institutional Animal Care and Use Committee (IACUC) of the National Taiwan University (Approval number: NTU-108-EL-00173) and the Taipei Zoo (Approval number: 10901). The peripheral blood samples from Bornean orangutans utilized in this study were obtained from a 23-year-old female orangutan housed at the Taipei Zoo, which had not conceived after reaching sexual maturity. The IACUC of the Taipei Zoo conscientiously scrutinized all sampling procedures to ensure the utmost ethical standards were upheld.

### 2.2 Derivation of bo-iPSCs

The bo-iPSCs were generated through the introduction of key reprogramming factors, including human *OCT4, KLF4*, *SOX2*, and *c-MYC* into PBMCs using non-integrating Sendai-virus, CytoTune-iPS 2.0 Sendai Reprogramming Kit (A16517, Thermo Fisher Scientific), according to the manufacturer’s instructions. Briefly, PBMCs were plated in a complete StemPro-34 (10639011, Thermo Fisher Scientific) medium containing cytokines for 4 days, including SCF, FLT-3 Ligand, IL-3, and IL-6, to expand the erythroblast population. Then, the PBMCs were transduced with the CytoTune 2.0 Sendai reprogramming vectors (MOI = 5), and the cytokines were removed from the StemPro-34 medium (Day 0). The Sendai viruses were removed from the culture medium on Day 1, and the reprogrammed PBMCs were cultured in the complete StemPro-34 medium. On Day 3, the reprogrammed PBMCs were switched to cytokine-free StemPro-34 medium for reprogramming. The iPSC-like colonies appeared within 9–25 days, and colonies with an ESC-like appearance were manually isolated based on morphology between Day 15 and Day 25. The cells were passaged with TrypLE express Enzyme (12605036, Thermo Fisher Scientific) every 4–5 days and incubated at 37°C in a humidified atmosphere containing 5% CO_2_.

### 2.3 Bo-iPSCs culture

Three bo-iPSC lines were obtained from reprogramming the PBMCs of Bornean orangutan and cultured in human ESC medium (hESM) on inactive mouse fibroblast cells (iMEFs) coated plates. Subsequently, all three bo-iPSC cell lines were adapted to a feeder-free culture system using Essential 8 Flex Medium Kit (A2858501, ThermoFisher) and Matrigel matrix (354230, Corning) for routine cell culture maintenance. Passaged bo-iPSCs every four to 5 days using TrypLE express Enzyme (12605036, Thermo Fisher Scientific). All cells were cultured at 37°C in a humidified atmosphere containing 5% CO_2_. Initially, a feeder-dependent culture approach employing a human embryonic stem cell medium (hESM) expanded the bo-iPSCs. To mitigate the variability stemming from fluctuations across different media batches and to establish a consistent, dependable culture milieu for bo-iPSCs, the transition was made to use the Essential 8 (E8) medium as an ensuing cultivation platform. The E8 medium represents a xeno-free and feeder-free substrate that facilitates human PSCs’ robust proliferation and amplification (Chen and Li, 2011).

### 2.4 Conversion of bo-iPSCs pluripotency applied with m4i/L/A media

The m4i/L/A media include a 50/50 mixture of DMEM/F12 (11320-032, Gibco) and Neurobasal (21103-049, Gibco), 1% N2 (17502-048, Gibco), 2% B27 (17504-044, Gibco), 20 ng/mL hLIF (300-05, Peprotech), 0.5X GlutaMAX (35050-061, Gibco), 1% NEAA (11140-050, Gibco), 0.1 mM 2-Mercaptoethanol (21985-023, Gibco), 1x Penicillin-Streptomycin (15140-122, Gibco), 50 ug/mL BSA (A3311, Sigma), 1 μM PD0325901 (1408, Axon), 1X Lipid Mixture (L0288, Sigma), 1 mM IM-12 (GSK3beta I, BML-WN102-0005, Enzo), 1 μM WH-4-023 (Src inhibitor, ENZ-CHM145-0010, Enzo), 10 μM Y-27632 (1254, Tocris), 20 ng/mL Activin A (AF-120-14E, Peprotech), 8 ng/mL bFGF (AF-100-18B, Peprotech), and 0.50% KSR (10828-028, Gibco). The E8 bo-iPSCs were dissociated into single cells with TrypLE express (12605010, Gibco) and re-plated on MEFs in E8 media with Y27632 (1254, Tocris) at a density of 200,000 cells per well in a 6-well plate. After 2 days, the media was changed to m4i/L/A media. Media was changed daily, and m-bo-iPSCs were passaged every 4 days at a ratio between 1:5. bo-iPSCs were preserved using Bambanker cryopreservation media (BB01, Nippon Genetics Co.) as the protocol described.

### 2.5 Karyotyping

The cells were incubated in KaryoMAX Colcemid Solution (15210040, Gibco) for 3 h. Subsequently, the cells were harvested and resuspended in a 75 mM KCl solution. Following a 30-min incubation at 37°C, 0.5 mL of fixative solution (methanol: acetic acid, 3:1 v/v) was added to the KCl solution, and centrifugation was performed at 300 g for 10 min. The supernatant was then removed, and 5 mL of cold fixative solution was added. The sample was incubated on ice for 30 min. These steps were repeated, and the resulting cell suspension was carefully applied to glass slides. Finally, the slides were stained with a Giemsa stain solution for 20 min in room temperature, and rinsed with tap water and airdry.

### 2.6 RT-PCR

Total RNA was isolated by TRIzol^®^ reagent (15596026, Invitrogen^™^) and treated with RNase-free DNase I (M6101, Promega, Madison, WI, USA) to remove genomic DNA. Treated RNAs were reverse-transcribed by random hexamer primers using the SuperScript III First-Strand Synthesis System (18080-051, Invitrogen). For conventional semi-quantitative PCR, cDNA (50 ng) was assayed at 30 s at 95°C, 30 s at 55°C, and 30 s at 72°C for 35 cycles by PCR machine (Biorad). PCR products were run on agarose gel electrophoresis and photographed by gel image system (UVP).

### 2.7 Quantitative real-time PCR

Total RNA was isolated using the TRIzol^®^ reagent (15596026, Invitrogen^™^) and Direct-zol RNA Miniprep Kits (R2052, ZYMO Research). The cDNA synthesis was performed from total RNA using FIREScript RT cDNA Synthesis Mix (062000100, Solis BioDyne). Real-time PCR was performed using SYBR Green qPCR Master Mix (4385616, Thermo Fisher Scientific) on the QuantStudio 5 Real-Time PCR System (Applied Biosystems). Gene expression was normalized to *GAPDH* and *RPL13A*.

### 2.8 Immunofluorescence staining

The cells affixed onto coverslips were fixed utilizing a 10% formaldehyde solution (MA-H121-08, Crespellano, Italy). Cells were subsequently permeabilized by utilizing 2% bovine serum albumin (BSA, A9647, MilliporeSigma) and 0.25% Triton-X-100 (X100, MilliporeSigma) within phosphate-buffered saline (PBS, IB3012, Omics Bio, Taipei, Taiwan) before being incubated with the primary antibodies overnight at 4°C. Secondary antibodies and DAPI were employed for 2 h at room temperature. OCT4 (1:150, MAB4401, Millipore), SOX2 (1:150, GTX101507, Genetex, Alton Pkwy Irvine, CA, USA), NANOG (1:500, ab21624, Abcam, Boston, MA, USA), SSEA4 (1:150, MAB4304, Millipore), KLF17 (sc-7392, Santa Cruz, Dalla, TX, USA), PAX6 (1:400, GTX113241, Genetex, Alton Pkwy Irvine, CA, USA), AFP (1:600, ST1673, Calbiochem, San Diego, CA, USA), and α-SMA (1:300, GTX60466, Genetex, Alton Pkwy Irvine, CA, USA) were utilized as primary antibodies, whereas Alexa donkey anti-rabbit 488 (A21206, Thermo), Alexa goat anti-mouse 647 (A221235, Thermo), Alexa goat anti-mouse 488 (A11029, Thermo), Alexa goat anti-rabbit 488 (A32731, Thermo), Alexa goat anti-mouse 594 (A11032, Thermo), and Alexa goat anti-rabbit 647 (A27040, Thermo) were employed as secondary antibodies. The laser-scanning confocal microscope (TCS SP5 II confocal microscope, Leica, Wetzlar, Germany) was employed to acquire images.

### 2.9 Teratoma assay

BALB/c Nu mice were purchased from LASCO Company and maintained in an IVC cage. The iPSCs cultured in E8 medium are trypsinized and intramuscularly injected into the hind leg 5 × 10^5^ per site. After 6 weeks, mice were sacrificed and the tumors were dissociated and fixed in PFA. Fixed samples were embedded and analyzed by hematoxylin and Eosin (H&E) staining.

### 2.10 RNA-seq

#### 2.10.1 RNA isolation, library preparation, and sequencing

Total RNA extraction was performed using Trizol^®^ Reagent (Invitrogen, USA), according to the manufacturer’s instructions. The purified RNA was quantified and assessed for quality using an ND-1000 spectrophotometer (Nanodrop Technology, USA) and a Bioanalyzer 2100 (Agilent Technology, USA) with the RNA 6000 LabChip kit (Agilent Technology, USA), respectively. The library was prepared by using SureSelect XT HS2 mRNA Library Preparation kit (Agilent, USA). Size selection was performed using AMPure XP beads (Beckman Coulter, USA). The RNA sequencing was performed on an Illumina Novaseq6000 platform, generating 2 × 150 bp paired-end sequencing reads. Data quality was assessed using Illumina Sequencing Analysis Viewer (SAV), and demultiplexing was performed using Bcl2fastq2 v2.20 software. The accession number for the bo-iPSCs RNA-Seq dataset is GSE235790.

#### 2.10.2 RNA-seq analysis

The raw fastq files were assessed using FastQC v0.11.9, and the results were summarized using MultiQC v1.13. Trimmomatic v0.39 was utilized for adapter removal and quality filtering of low-quality bases. The trimmed and untrimmed reads were then aligned to the *Pongo abelii* Susie_PABv2 reference genome with Ensembl v108 annotations using STAR v2.7.10b. The mapping rate for all bo-iPSC samples ranged from 77.2% to 78.8%. The featureCounts command line was employed to count the mapped reads for genes using the Rsubread v2.0.1 package. Differential gene expression analysis was conducted using DESeq2 v1.38.3, with a *p*-value cut-off of 0.05 and an absolute log2 fold change (log2FC) cut-off of 2.00, indicating statistical significance. To compare our RNA-Seq dataset with published RNA-Seq datasets (Accession number: GSE135991 (Yu et al., 2021), GSE75868 (Theunissen et al., 2016), E-MTAB-4461 (Guo et al., 2016)) representing different pluripotent statuses, we followed the same bioinformatic workflow and visualized the results using Principal Coordinate Analysis (PCoA) with the phyloseq v1.42.0 package.

#### 2.10.3 Functional enrichment analyses of DEGs

The DEGs were analyzed with the DAVID bioinformatics resources (Huang et al., 2009; Sherman et al., 2022) (https://david.ncifcrf.gov/) for gene ontology (GO) biological processes (BP), cellular components (CC), and molecular functions (MF) as well as the KEGG pathway, using the default gene list of *Pongo abelii* as the background. The functional enrichment analysis results are presented using Prism (GraphPad Software, La Jolla, CA, USA) in the form of box plots.

### 2.11 RNA ISH

Cells were collected and resuspended in culture medium (E8 and m4i/L/A). Adjust the cell concentration to 1 × 10^6^ per mL. Spread the cells to the Superfrost plus microscope slides (1255015, Fisher Scientific) for adhesion. Allow the cells to adhere for 10 min and wash with DPBS. Afterward, quickly proceeded to the fixation and hybridization steps following the manufacturer’s protocols. The HUWE1 FISH probe (1104261C1, ACDBio) was labeled with Opal 620 dye. Images were acquired using a LEICA TCS SP8X STED confocal microscope. A minimum of 60 nuclei were analyzed for each condition to quantify the expression of HUWE1**.**

### 2.12 Embryoid body (EB) formation

To ensure precise collection of the predetermined quantity of m-bo-iPSCs, a centrifugation procedure at 100 x g for 3 min was conducted to accumulate cells at the bottom, generating the desired size of EBs. Initially, m-bo-iPSCs underwent single-cell dissociation using TrypLE. Subsequently, these cells were cultured in a well (5,000 cells in each) on an ultra-low attachment plate through centrifugation. Then, aggregated 5,000 cells were cultured in EB media [DMEM/F12 supplemented with 20% FBS, 1X GlutaMax, 1X MEM NEAA, 0.1 mM β-mercaptoethanol, and 1% penicillin-streptomycin] supplemented with 10 μM Y-27632 (Dong et al., 2020) in ultra-low attachment multiple-well plate (651970, Corning Coster). Media changes were implemented every 2 days. Following 12 days of cultivation, the EBs were harvested for downstream analysis.

### 2.13 Statistical analysis

All data were presented as means ± standard error of the mean (SEM) and evaluated by ordinary one-way ANOVA or unpaired *t*-test with Welch’s correction using Prism (GraphPad Software, La Jolla, CA, USA). When *p* < 0.05 were considered to be s statistically significant.

## 3 Results

### 3.1 Generation of the bornean orangutan iPSCs by non-integrating sendai viral-mediated reprogramming

**FIGURE 1:**
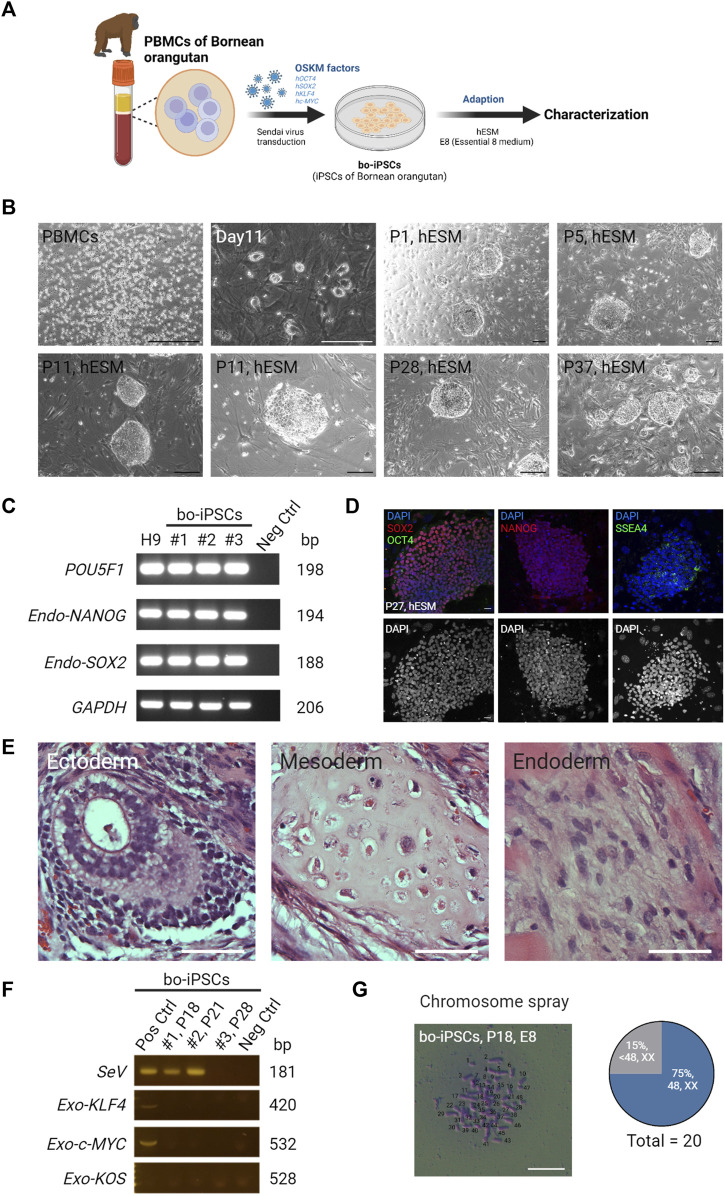
Generation and characterization of bo-iPSCs in hESM. **(A)** A schematic diagram depicting the derivation and *in vitro* maintenance process of bo-iPSCs. **(B)** Representative bright-field images showing Bornean orangutan PBMCs and round colony morphologies of bo-iPSCs cultured under hESM for long-term *in vitro* maintenance. Scale bars, 200 μm. **(C)** RT-PCR gel image demonstrating the expression of endogenous pluripotency markers (POU5F1, NANOG, and SOX2) in three bo-iPSC cell lines, namely, cell line 1 (#1, passage 18), cell line 2 (#2, passage 21), and cell line 3 (#3, passage 28), compared to H9 hESCs and a negative control (fibroblast cells). **(D)** Representative immunofluorescence images (upper panel) of bo-iPSCs cultured under hESM. Red indicates SOX2 and NANOG; green indicates OCT4 and SSEA4; blue indicates DAPI; the lower panel displays the DAPI staining in an 8-bit format. Scar bar, 25 μm. **(E)** Representative H&E images demonstrating the ability of bo-iPSCs cultured under E8 to differentiate into all three germ layers in teratoma formation. Scale bar, 50 μm. **(F)** RT-PCR showing the presence of Sendai virus genome (SeV) and exogenous OSKM genes (KOS, KLF4, and c-Myc) is detected in early passages of bo-iPSC cell line 1 (#1, passage 18) and cell line 2 (#2, passage 21), but is cleared in bo-iPSC cell line 3 (#3, passage 28). **(G)** Bright field image of chromosome spread revealing E8 bo-iPSCs with the characteristic display of normal chromosome numbers. Scale bar, 100 μm. Quantitative analysis of twenty E8 bo-iPSC cells demonstrates that 75% of the bo-iPSCs exhibited normal chromosome numbers.

Peripheral blood mononuclear cells were obtained from a captive female Bornean orangutan at Taipei Zoo. These PBMCs were reprogrammed by Sendai virus-mediated transduction of human OSKM factors (Figure 1A), and three bo-iPSC cell lines were generated (Supplementary Figure S1A). Flat, well-defined colonies appeared 11–15 days after transfection (Figure 1B). Following cell passaging, bo-iPSC colonies retained the dome-shaped morphology (Figure 1B) within the human embryonic stem cell (hESC) medium culture system. Upon transitioning bo-iPSCs into the E8 culture system, the bo-iPSC colonies transitioned to a flatter appearance (Supplementary Figure S1B). These colonies were positive for alkaline phosphatase (AP) staining (Supplementary Figure S1C). We next determined the transcription patterns of key pluripotency-associated genes of the bo-iPSCs (Figure 1C, Supplementary Figure S1D) and translational levels (Figure 1D, Supplementary Figure S1E). These cells displayed the expression of core pluripotency-associated genes, specifically *POU5F1, SOX2*, and *NANOG*, through qRT-PCR analysis. Immunostaining data showed that the bo-iPSCs expressed major pluripotency-related markers, including OCT4, SOX2, NANOG, and SSEA4. The *in vivo* teratoma assay confirmed that the bo-iPSCs are capable of differentiating to all three germ layers (Figure 1E, Supplementary Figure S1F). In addition, we used RT-PCR to detect if there are any residual Sendai virus genome and exogenous pluripotent genes in these bo-iPSCs at passage 28, and results indicated none (Figure 1F). Also at this passage, we conducted the chromosome spray analysis and revealed that 75% of assessed colonies (n = 20) had normal chromosome numbers (Figure 1G).

Collectively, the bo-iPSCs were successfully derived from Bornean orangutans, which expressed core pluripotency-related markers genes, and possessed high differentiation capacity into all three germ layers.

### 3.2 Adaptation of bo-iPSCs to the m4i/L/A culture system

Prior studies have suggested that most primate iPSCs derived by the OSKM factors are primed PSCs (Nichols and Smith, 2009; Weinberger et al., 2016). Converting primed human PSCs to a naive state by using different combinations of specific molecular inhibitors and growth factors supplemented in the medium to modulate and maintain the naive pluripotency has been reported (Theunissen et al., 2014; Guo et al., 2016; Bredenkamp et al., 2019). In our pilot study, we applied 5i/L/A medium to bo-iPSCs culture, but observed pronounced apoptotic morphology in the bo-iPSCs when cultured in a 5i/L/A medium, which resulted in an inability to sustain their *in vitro* viability (Supplementary Figure S2A). However, upon discontinuing the BRAF inhibitor SB590885, the bo-iPSCs exhibited improved survival. Building upon these findings, we subtracted SB590885 from the original 5i/L/A recipe, and used this modified 4i/L/A (m4i/L/A) medium to culture the OKSM factor derived bo-iPSCs, with the objective to convert these cells to naive state.

**FIGURE 2:**
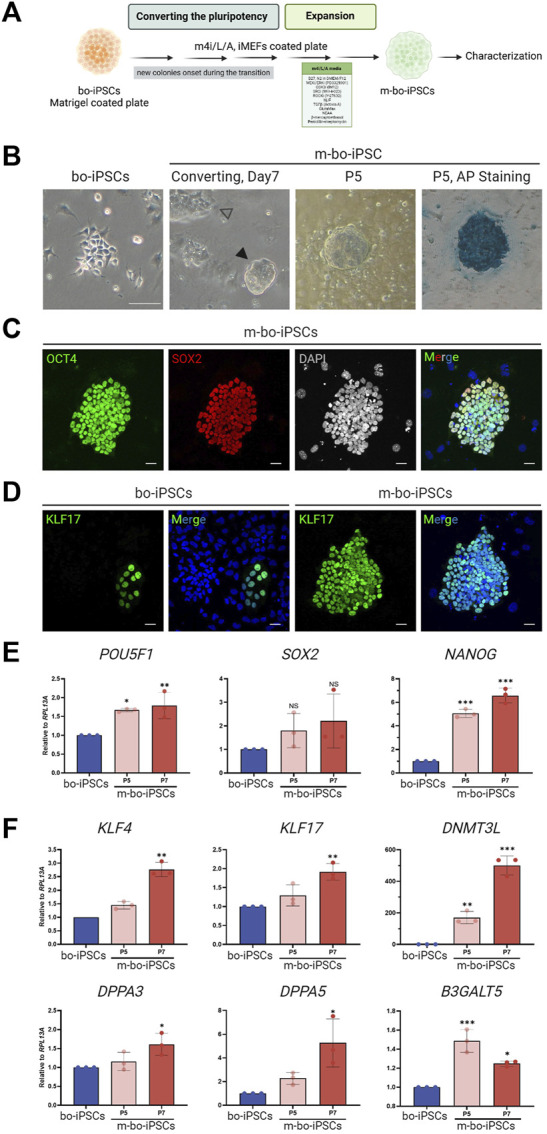
The pluripotent characteristics of bo-iPSCs cultured in m4i/L/A Medium. **(A)** Schematic representation of the process of bo-iPSCs adaptation from E8 to m4i/L/A medium, with the green box denoting the key components used in m4i/L/A medium. **(B)** Representative bright-field images illustrating the change in bo-iPSCs colony morphology during media adaptation from E8 to m4i/L/A, along with an alkaline phosphatase (AP) staining image of bo-iPSCs cultured in m4i/L/A (passage 5) showing positive AP activity. The black arrowhead indicates the domed-shaped colony, while the white arrowhead denotes the appearance of a flattened colony. Scale bars, 100 μm. **(C,D)** Representative immunofluorescence images of m-bo-iPSCs cultured under m4i/L/A at passage 5, demonstrating the sustained expression of OCT4 and SOX2 **(C)**, as well as the induction of KLF17 of m-bo-iPSCs **(D)** by the m4i/L/A system in comparison to bo-iPSCs cultured under E8 at passage 33. Red indicates SOX2; green indicates OCT4 and KLF17; blue indicates DAPI. The gray colony image represents the 8-bit format of DAPI staining. Scar bar, 25 μm. **(E,F)** qRT-PCR analysis revealed significant differences in the expression levels of core pluripotent genes, POU5F1 and NANOG **(E)**, and several pluripotency regulating markers in m-bo-iPSCs **(F)**. RPL13A was used as the internal control. Biological replicates, n = 3. Asterisks indicate *p < 0.05, **p < 0.01, and ***p < 0.001.

The m4i/L/A (Figure 2A) contained a GSK3β inhibitor IM-12 that is used to activate the WNT signaling pathway, while the other three inhibitors PD0325901, WH-04-023, and Y-27632 are used to repress the MAPK/ERK, SRC, and Rho/ROCK pathways, respectively.

The bo-iPSCs were seeded onto a culture plate with iMEF feeder cells cultivated in m4i/L/A medium. Through regular cell passaging every 4–5 days, we observed the emergence of small, dome-shaped colonies that gradually became the predominant population in passage 3. These adapted bo-iPSCs under the m4i/L/A condition are named m-bo-iPSCs (Figure 2A).

The m-bo-iPSCs, in addition to the dome-shaped colony morphology (Figure 2B), were alkaline phosphatase positive and expressed OCT4 and SOX2 (Figure 2C); moreover, the KLF17 pluripotent markers are dominantly expression compared to bo-iPSCs (Figure 2D), as assayed by immunostaining. The qRT-PCR results revealed high level expression of core pluripotent factors *POU5F1* and *NANOG* (Figure 2E), as well as several additional pluripotent markers, including *KLF17*, *DNMT3L*, and *DPPA3/*5, when compared to bo-iPSCs (Figure 2F). Interestingly, the other pluripotent marker gene *B3GALT5* showed a significantly reduced trend of transcription in m-bo-iPSCs along with passaging (Figure 2E).

These results demonstrated that the bo-iPSCs can adapt to the m4i/L/A culture system, and the resultant m-bo-iPSCs have different molecular and cellular pluripotent characteristics from the bo-iPSCs.

### 3.3 Distinct transcriptome profile and X chromosome status of m-bo-iPSCs

**FIGURE 3:**
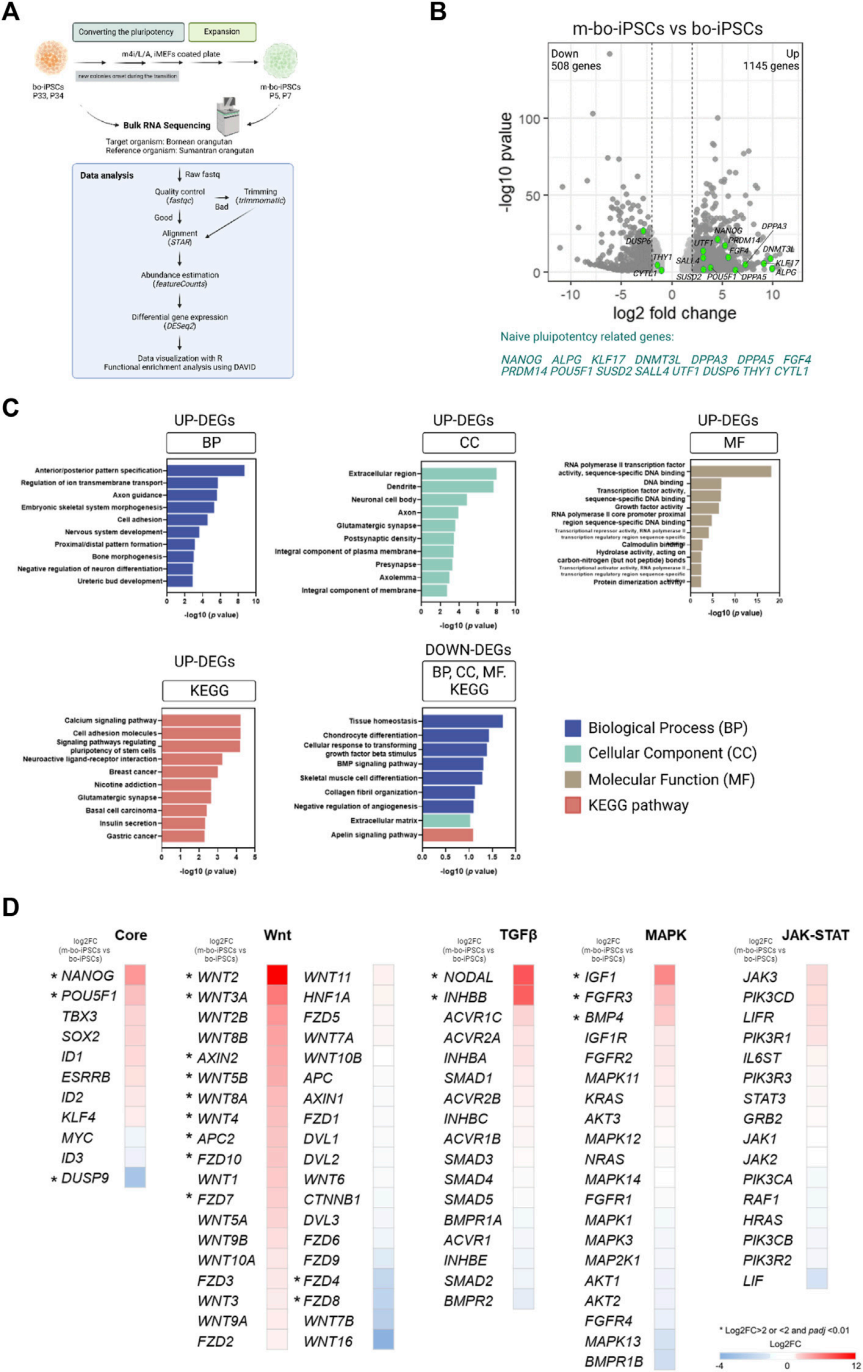
The transcriptome profile of the m4i/L/A-cultured and E8-cultured bo-iPSCs. **(A)** The flow diagram outlining the process of bulk RNA sequencing and subsequent analysis of bo-iPSCs and m-bo-iPSCs. **(B)** Volcano plot displaying the log2 fold change (*X*-axis) between m-bo-iPSCs and bo-iPSCs. Among the differentially expressed genes (DEGs), 1145 genes were significantly upregulated, and 508 genes were considerably downregulated in m-bo-iPSCs (defined as those with a log2 fold change >1 and wherein -log10 *p* > 1.3). The green dots indicate the genes related to naive pluripotency regulating in human studies. Biological replicates, n = 2. **(C)** Box plots presenting the top ten gene ontology (GO) terms and KEGG pathways of the significantly up- and downregulated DEGs involved in the adaptation of bo-iPSCs from E8 to m4i/L/A. BP: biological process, CC: cellular component, and MF: molecular function. **(D)** Heatmap revealing the impact of the m4i/L/A system on core pluripotent signaling pathway activity, with the Wnt and TGFβ signaling pathways being predominantly active. The symbol “*” indicates a significant difference.

We conducted RNA-sequencing (RNA-seq) analysis to characterize the transcriptional profiles of m-bo-iPSCs and the bo-iPSCs, (Figure 3A). Given the high genomic similarity between Bornean and Sumatran orangutans (Chen and Li, 2001; Locke et al., 2011), we used the Sumatran orangutan as the reference organism for the alignment in the RNA-seq analysis pipeline. The mapping rate obtained by STAR ranged from 75.8% to 78.8%.

The unsupervised hierarchical clustering and principal component analysis (PCA) revealed that m-bo-iPSCs are transcriptionally different from the bo-iPSCs (Supplementary Figure S2B, S2C). Notably, the volcano plot revealed that several transcripts associated with self-renewal and pluripotency were significantly upregulated in m-bo-iPSCs vs. bo-iPSCs, defined as those with a log2 fold change [log2FC] > 1 and wherein -log10 *p*-value >1.3, which included *NANOG, POU5F1, ALPG, UTF1, SALL4*, *DPPA3, DPPA5, KLF17*, and *DNMT3L* (Figure 3B, Supplementary Figure S2D).

Next, we conducted an enrichment analysis of the differentially expressed genes (DEGs) by the Gene Ontology (GO) terms and the Kyoto Encyclopedia of Genes and Genomes (KEGG) pathway analysis.

We first looked at the upregulated DEGs in the m-bo-iPSCs. The GO analysis revealed differences between the m-bo-iPSCs and the bo-iPSCs in biological processes such as anterior/posterior pattern specification (GO:0009952), regulation of ion transmembrane transport (GO:0034765), and axon guidance (GO:0007411) (Figure 3C). In terms of cellular components, the significantly upregulated differentially expressed genes (DEGs) in the m-o-iPSCs had a significant enrichment in the extracellular region (GO:0005576), dendrites (GO:0030425), and neuronal cell bodies (GO:0043025) (Figure 3C). The top three molecular functions associated with the upregulated DEGs in the m-bo-iPSCs were RNA polymerase II transcription factor activity, sequence-specific DNA binding (GO:0000981), DNA binding (GO:0003677), and transcription factor activity, sequence-specific DNA binding (GO:0003700) (Figure 3C). Moreover, Kyoto Encyclopedia of Genes and Genomes (KEGG) pathway analysis demonstrated that upregulated DEGs in the m-bo-iPSCs were enriched in the calcium signaling pathway, cell adhesion molecules, and signaling pathways regulating pluripotency of stem cells (Figure 3C).

We then looked at the downregulated DEGs in the m-bo-iPSCs. GO analysis revealed differences between the m-bo-iPSCs and the bo-iPSCs in several key biological processes, including tissue homeostasis (GO:0001894), chondrocyte differentiation (GO:0002062), and cellular response to transforming growth factor beta stimulus (GO:0071560) (Figure 3C). With regard to cellular component in the GO terms analysis, the downregulated DEGs in the m-bo-iPSCs were associated with the extracellular matrix (GO:0031012). The KEGG pathway analysis further suggested that the downregulated DEGs in the m-bo-iPSCs were related to the apelin signaling pathway (Figure 3C).

We then analyzed the signaling pathways essential for pluripotency maintenance and self-renewability in the bo-iPSCs and the m-bo-iPSCs. These pathways encompass the core pluripotency genes and those associated with Wnt, TGFβ, MAPK, and JAK-STAT signaling cascades. Compared to the bo-iPSCs, several genes in the Core pluripotency pathways, including, Nanog, Pou5f1, and Dusp9, were significantly upregulated. Robust activation of more than 10 genes, including Wnt2 and Wnt3A, in the Wnt pathway in the m-bo-iPSCs were revealed (Figure 3D). In the TGFβ pathway, Nodal and Inhbb were expressed at higher levels in the m-bo-iPSCs than in the bo-iPSCs. In the MAPK pathway, Igf1, FGFR3, and BMP4 were expressed at higher levels in the m-bo-iPSCs than in the bo-iPSCs. Interestingly, none of the genes in the JAK-STAT pathway had significantly different expression levels between the m-bo-iPSCs and the bo-iPSCs (Figure 3D).

In sum, the RNA-seq data and the functional enrichment assays revealed that the m4i/L/A culture system activated the WNT signaling pathway efficiently to drive the bo-iPSCs towards m-bo-iPSCs with a distinct set of molecular pluripotency features.

### 3.4 X chromosome activation status in the bo-iPSCs and the m-bo-iPSCs

One signature of naive state PSCs is that both X chromosomes are in the activated state (XaXa), whereas in the primed state PSCs, there is only one activated X chromosome (XaXi) (Takahashi et al., 2018).

**FIGURE 4:**
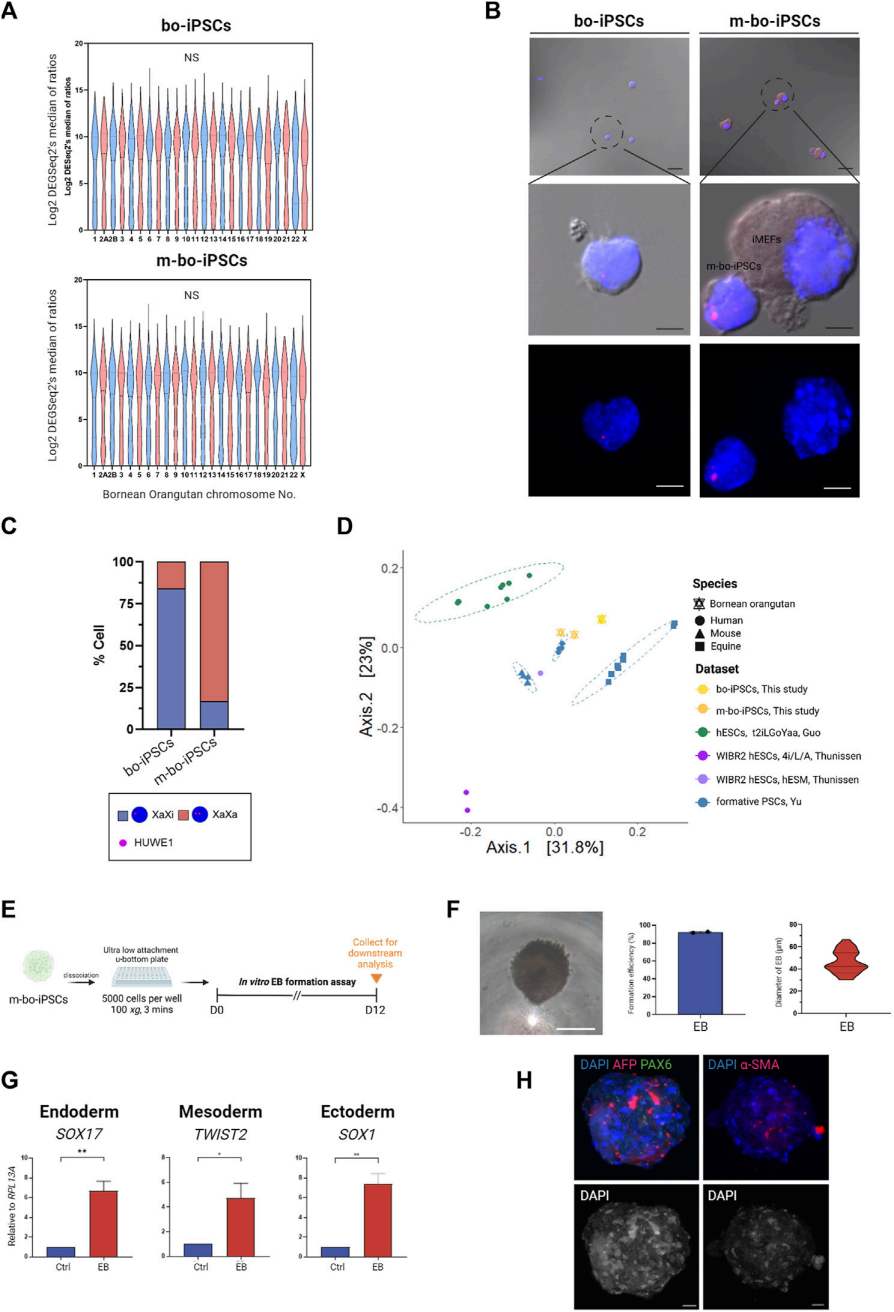
Characterization of m-bo-iPSCs. **(A)** Violin plots illustrate no significant difference in the gene expression levels of each chromosome between bo-iPSCs and m-bo-iPSCs. **(B)** Representative merged bright-field and fluorescence RNA ISH (top) and fluorescence RNA ISH (bottom) images revealing distinct X chromosome activation status of bo-iPSCs and m-bo-iPSCs, detected by HUWE1 nascent transcripts (red) in DAPI-stained nuclei (blue). Scale bar, 10 μm. **(C)** Bar plot quantification of the RNA ISH patterns in bo-iPSCs and m-bo-iPSCs, demonstrating a higher proportion of bi-allelic HUWE1 expression in m-bo-iPSCs. **(D)** PCoA plot of RNA-seq datasets depicting the pluripotency characteristics of the mouse (mESC, XPSC, and miPSCs), human (mESC, XPSC, and miPSCs), horse (FTW-eqESC and FTW-eqiPSC), bo-iPSCs and m-bo-iPSCs samples. **(E)** Schematic diagram outlining the experimental design for inducing spontaneous *in vitro* EB formation of m-bo-iPSCs. **(F)** The representative bright-field image displays the morphology of EB from m-bo-iPSCs. Scale bar, 25 μm. Box plots showing the high EB formation efficiency in m-bo-iPSCs, and a violin plot representing the diameter distributions of EB. **(G)** qRT-PCR results demonstrating significant expression levels of three germ layer markers in EB compared to the m-bo-iPSCs before *in vitro* differentiation (used as control). Endoderm: SOX17, mesoderm: TWIST2, and ectoderm: SOX1. RPL13A served as the internal control. Biological replicates, n = 2. Asterisks indicate *p < 0.05, **p < 0.01, and ***p < 0.001. **(H)** Representative immunofluorescence images of EB expressing markers of all three germ layers: ectoderm: PAX6 (green), endoderm: AFP (red), mesoderm: α-SMA (red), with nuclei stained in DAPI (blue). Gray colony image represents the 8-bit format of DAPI staining. Scale bar, 25 μm.

To gain knowledge on the effect of the m4i/L/A system on the X chromosome activation status in m-bo-iPSCs, we looked at an X-linked gene, HUWE1. The transcription levels of Huwe1 are similar between the bo-iPSCs and m-bo-iPSCs (Figure 4A), suggesting that the culture condition did not affect the transcription level of this gene. Next, we conducted RNA *in situ* hybridization (RNA-ISH) probing HUWE1. Intriguingly, the RNA-ISH data revealed that in the bo-iPSCs, HUWE1 (Figures 4B,C) displayed a highly mono-allelic expression pattern, whereas in the m-bo-iPSCs, most cells displayed a bi-allelic expression pattern of Huwe1 which suggests successful X-chromosome reactivation in the m-bo-iPSCs.

We then conducted a comparative analysis using RNA-seq datasets to evaluate the pluripotent state of bo-iPSCs cultured in E8 and m4i/L/A mediums. Our hypothesis is that the m4i/L/A adaptation process will convert the bo-iPSCs towards the naive state.

We included several published datasets of various pluripotent state cell lines in this comparative analysis, which included naive human hESCs derived from different cocktail mediums (4i/L/A (GSE75868 (Theunissen et al., 2016)) and t2iLGÖYaa (E-MTAB-4461 (Guo et al., 2016))), formative human/mouse/equine PSCs (GSE135991 (Yu et al., 2021)), and primed human hESCs (GSE75868 (Theunissen et al., 2016)).

The principal coordinate analysis (PCoA) plot (Figure 4D) indicate that m-bo-iPSCs clustered between t2iLGÖYaa naive hESCs and formative human iPSCs, notably distinct from the primed hESCs. In contrast, the E8 bo-iPSCs grouped closely with the formative equine PSCs rather than the human formative or primed PSCs groups.

Collectively, these results demonstrate that the bo-iPSCs and m-bo-iPSCs have distinctly different cellular and molecular characteristics.

### 3.5 m-Bo-iPSCs have spontaneous differentiation capacity to form three germ layers

To assess the differentiation capacity of the m-bo-iPSCs, we employed the *in vitro* embryoid body formation assay (Figure 4E). EBs emerged from greater than 90% of colonies, and exhibited compacted appearances (Figure 4F). The sizes of the derivative EBs ranged from 30.2 to 66.5 μm on Day 7 (Figure 4F). qRT-PCR analysis was performed to examine the expression of key germ layer markers: SOX17 for endoderm, TWIST2 for mesoderm, and SOX1 for ectoderm. As expected, the EBs derived from these m-bo-iPSCs expressed markers for all three germ layers (Figure 4G). Immunostaining assays confirmed the expression of all three germ lineage markers (PAX6 for ectoderm, AFP for endoderm, and α-SMA for mesoderm) in the EBs derived from m-bo-iPSCs (Figure 4H).

These data demonstrate that the m-bo-iPSCs possess the differentiation capacity to all three germ layers.

## 4 Discussion

Recently, the utilization of iPSCs in conservation efforts for endangered species has gained momentum, exemplified by studies on the Northern white rhinoceros (Zywitza et al., 2022), avian species (Katayama et al., 2022), and Grevy’s zebra (Endo et al., 2022). The derivation of orangutan iPSCs has also been reported but thus far been limited to a single species of *Pongo* genus, *Pongo abelii* (also known as Sumatran orangutans) (Ramaswamy et al., 2015; Geuder and Lucas, 2021). In this work, we report the successful generation of o-iPSCs from another species of *Pongo* genus, the *Pongo pygmaeus* (generally acknowledged as Bornean orangutan). Our work demonstrates the feasibility of adapting the iPSC technology for the preservation of Bornean orangutan genetics.

The o-iPSCs obtained from Bornean orangutans can be cultured extensively, surpassing 30 passages while still maintaining pluripotency and chromosomal integrity. These bo-iPSCs may be used for somatic cell nuclear transfer (SCNT), differentiation into gametes (i.e., oocytes or sperm), or for PSC cell-based embryo injections upon technology maturation, to produce Bornean orangutan offspring. Thus, our work expands the repertoire of orangutan iPSCs and represents the first step towards a tangible Bornean orangutan preservation strategy by advanced biotechnology.

The current work sheds light on the pluripotency regulation in primate pluripotent stem cells. We adapted the bo-iPSCs in a naive-state-promoting culture condition m4i/L/A through promoting the WNT pathway. The derivative m-bo-iPSCs displayed characteristic traits of akin to the human naive PSCs at both the cellular and molecular levels (Nichols and Smith, 2009; Smith, 2017; Pera and Rossant, 2021). Further, we demonstrated that the female m-bo-iPSCs displayed X chromosome reactivation, as indicated by the bi-allelic expression of an X-linked gene *HUWE1*, a key indication of the naive status of these cells based on knowledge gained from human PSCs (Sahakyan et al., 2017; Vallot et al., 2017). Our work thus suggests key roles of the WNT pathway in primate PSCs’ naive pluripotency. From the practical point of view, the m-bo-iPSCs may serve better than the o-iPSCs for animal preservation purposes. Due to the differences in primordial germ cells (PGC) specification across species (Ginsburg, Snow, and McLaren, 1990; Sasaki et al., 2016; Kobayashi et al., 2017), whether the evidence from experimental models in different species fits in orangutans’ PGC specification remains unclear (Ohinata et al., 2009; Hayashi et al., 2011; Sasaki et al., 2015; Seita et al., 2023). Deriving the PGC-like cells (PGCLCs) from m-bo-iPSCs will highlight the further specific application in species preservation and broaden the knowledge of PGC specification across species.

In conclusion, our study has successfully generated Bornean orangutan iPSCs, thereby increasing the species diversity in the repertoire of orangutan iPSCs. We found that the m4i/L/A culture system promoted bo-iPSCs towards the naive pluripotency state through the activation of the WNT pathway. These findings contribute to the application of advanced biotechnology in preserving endangered species and shed light on the pluripotency regulation in primate species.

## Data Availability

The datasets presented in this study can be found in online repositories. The names of the repository/repositories and accession number(s) can be found in the article/Supplementary Material.
