# Supplementary figures and images for: Generation of induced pluripotent stem cells from Bornean orangutans
Source: Front Cell Dev Biol. 2024 Jan 5;11:1331584. doi: 10.3389/fcell.2023.1331584 (PMC10797036; doi:10.3389/fcell.2023.1331584)

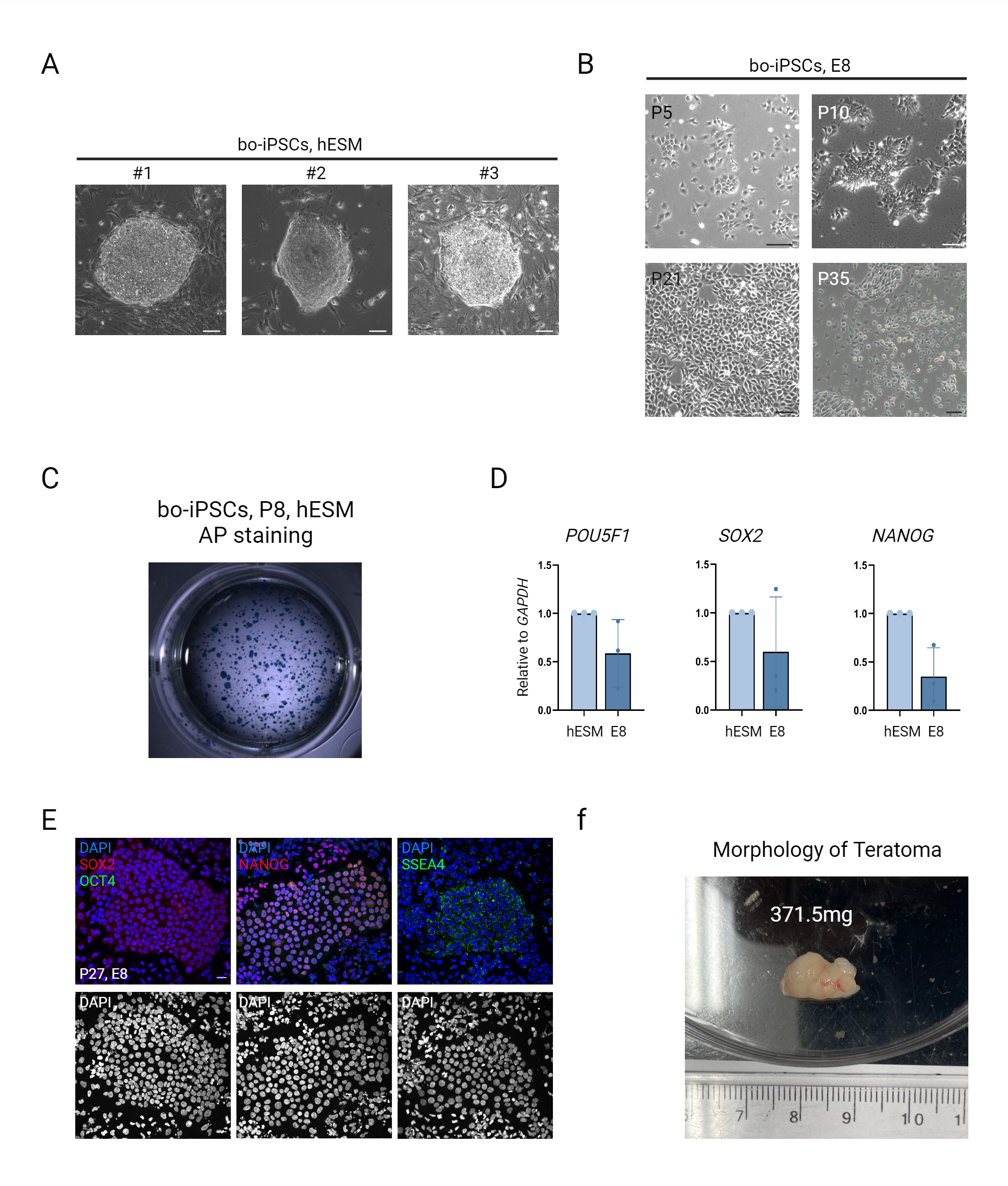

Supplement: Supplementary file 1 [file Image1.JPEG]

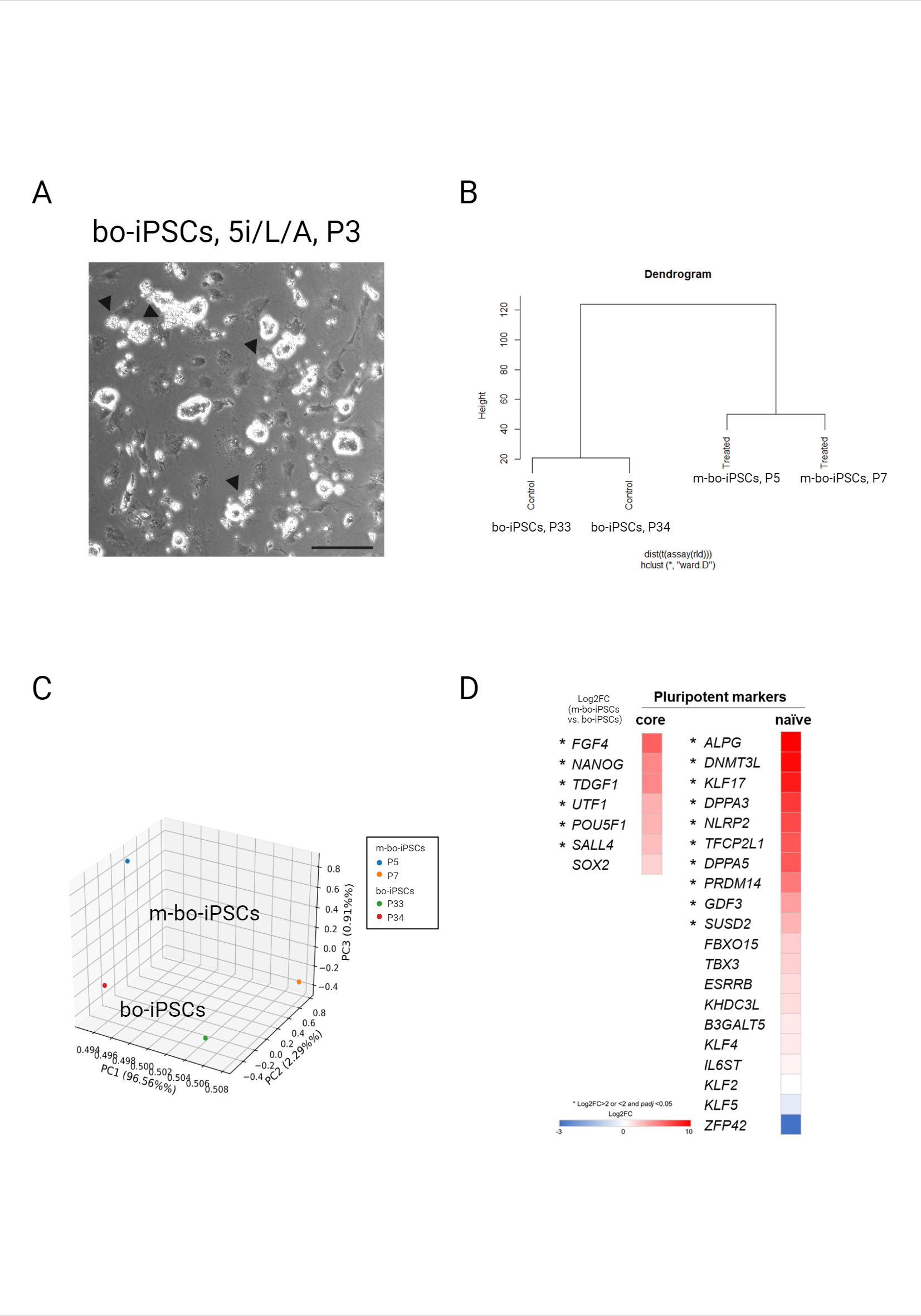

Supplement: Supplementary file 2 [file Image2.JPEG]
