# Supplementary material for: Generation of induced pluripotent stem cells from Bornean orangutans
Source: Front Cell Dev Biol. 2024 Jan 5;11:1331584. doi: 10.3389/fcell.2023.1331584 (PMC10797036; doi:10.3389/fcell.2023.1331584)
Supplement: Supplementary file 3 [file DataSheet1.docx]

Supplementary Material

**
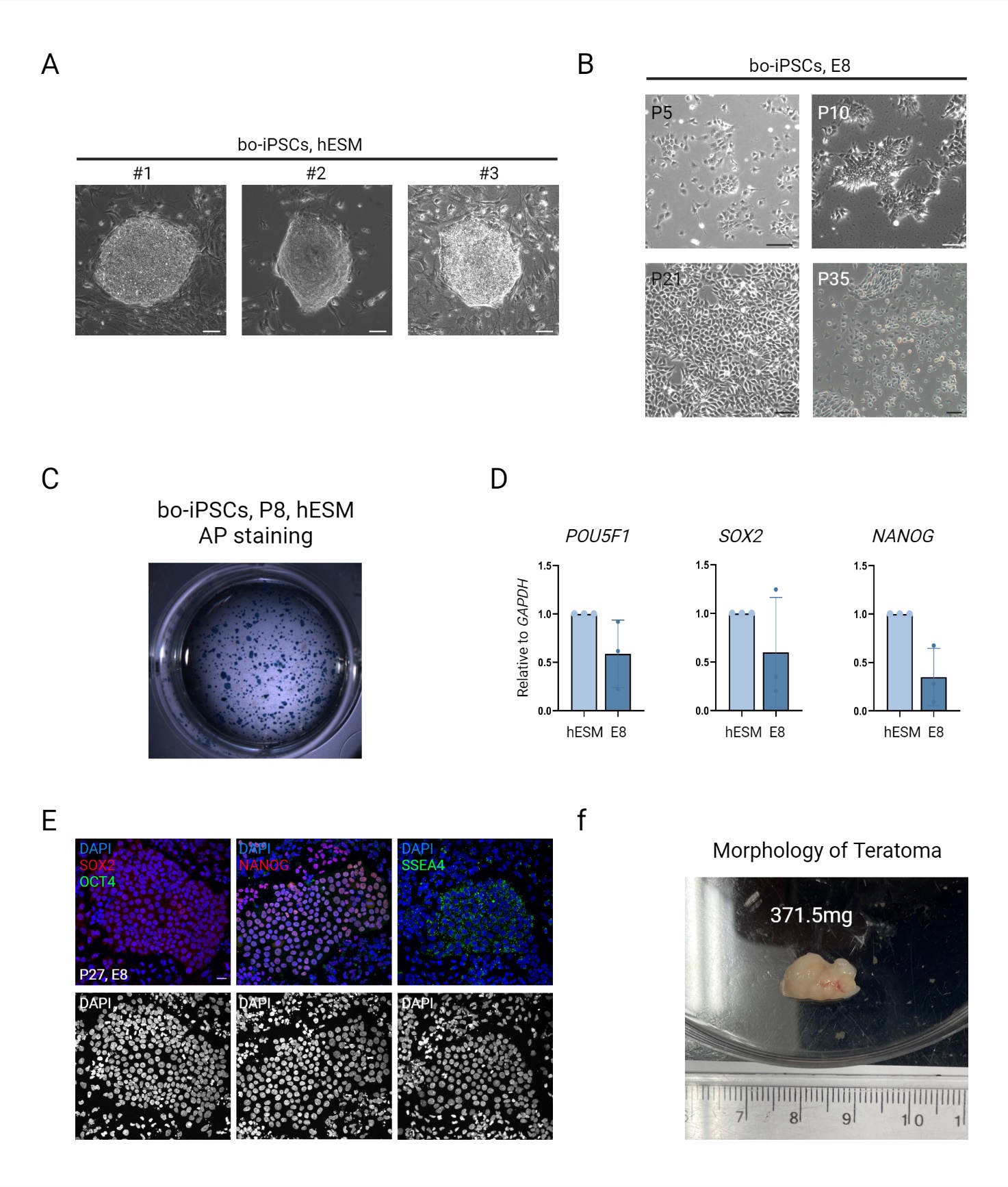
**

**Supplementary FIGURE 1. Three Bornean orangutan induced pluripotent stem cell lines, related to Figure 1. (A)** Representative phase contrast images of three bo-iPSC colonies. Scale bar=100 μm. **(B)** Alkaline phosphatase (AP) staining image of bo-iPSCs culture in hESM (passage 8) showing positive AP activity of o-iPSCs. **(C)** Representative phase contrast images of E8-cultured bo-iPSC colonies for long-term *in vitro* maintenance. Scale bar, 200 μm. **(D)** qRT-PCR analysis reveals no significant differences in the expression levels of core pluripotent genes, POU5F1, SOX2, and NANOG, between hESM-cultured and E8-cultured bo-iPSCs. **(E)** Representative immunofluorescence images (upper panel) of bo-iPSCs cultured under E8. Red indicates SOX2 and NANOG; green indicates OCT4 and SSEA4; blue indicates DAPI. The lower panel displays the DAPI staining in an 8-bit format. Scar bar, 25 μm. **(F)** The appearance of teratoma derived from intramuscularly injecting the E8-cultured bo-iPSCs into BALB/c Nu mice.

**
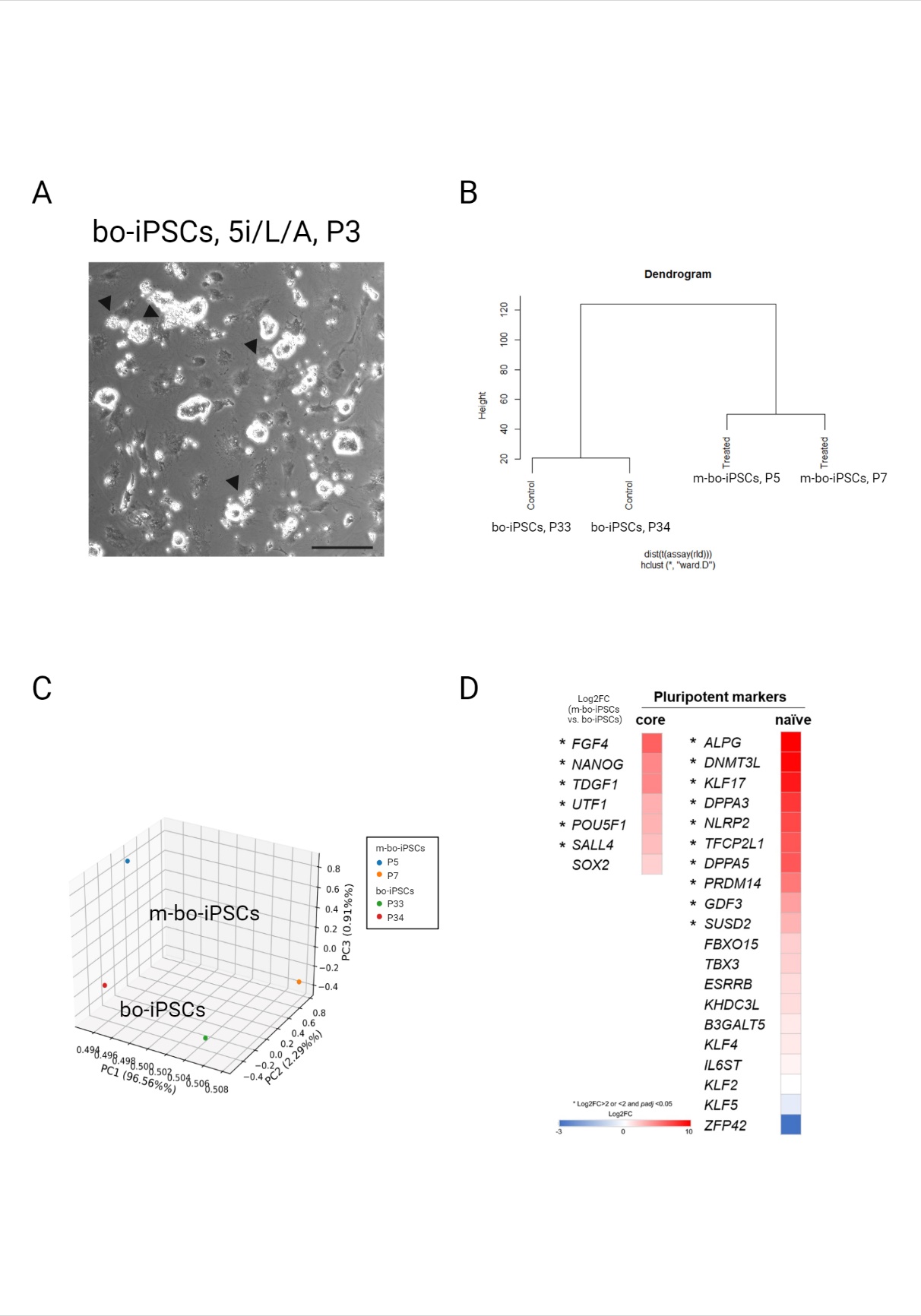
**

**Supplementary FIGURE 2. The morphology of bo-iPSCs cultured in 5i/L/A and RNA-seq data of bo-iPSCs and m-bo-iPSCs, related to Figure 3. (A)** Representative bright-field images illustrating the bo-iPSCs apoptotic colony morphology during media adaptation from E8 to 5i/L/A. Black arrowhead indicating the apoptotic colony. Scale bars, 200 μm. **(B)** Unsupervised hierarchical clustering analysis reveals distinct bulk RNA-sequencing results in the bo-iPSCs and m-bo-iPSCs samples, leading to the formation of two distinct clusters. Biological replicates, n = 2. **(C)** PCA plot of bulk RNA-seq data from bo-iPSCs and m-bo-iPSCs samples. **(D)** Heatmap illustrating the Log2 fold change (Log2FC) between bo-iPSCs and m-bo-iPSCs, highlighting the differential expression of core and naive pluripotent markers as determined by bulk RNA-seq analysis.
